# Influence of body mass index on outcomes after minimal-access aortic valve replacement through a J-shaped partial upper sternotomy

**DOI:** 10.1186/s13019-016-0467-2

**Published:** 2016-04-27

**Authors:** Metesh Acharya, Leanne Harling, Marco Moscarelli, Hutan Ashrafian, Thanos Athanasiou, Roberto Casula

**Affiliations:** Department of Cardiothoracic Surgery, Hammersmith Hospital, London, UK; The Department of Surgery and Cancer, 10th Floor QEQM Building, St Mary’s Hospital, Praed St., London, W2 1NY UK

**Keywords:** Aortic valve replacement, Minimally invasive, Minimal access, Cardiac surgery

## Abstract

**Background:**

Minimal-access aortic valve replacement (MAAVR) may reduce post-operative blood loss and transfusion requirements, decrease post-operative pain, shorten length stay and enhance cosmesis. This may be particularly advantageous in overweight/obese patients, who are at increased risk of post-operative complications. Obese patients are however often denied MAAVR due to the perceived technical procedural difficulty. This retrospective analysis sought to determine the effect of BMI on post-operative outcomes in patients undergoing MAAVR.

**Methods:**

Ninety isolated elective MAAVR procedures performed between May 2006–October 2013 were included. Intra- and post-operative data were prospectively collected. Ordinary least squares univariate linear regression analysis was performed to determine the effect of BMI as a continuous variable on post-operative outcomes. One-way ANOVA and Chi-squared testing was used to assess differences in outcomes between patients with BMI <25 (*n* = 36) and BMI ≥25 (*n* = 54) as appropriate.

**Results:**

There was no peri-operative mortality, myocardial infarction or stroke. Univariate regression demonstrated longer cross-clamp times (*p* = 0.0218) and a trend towards increased bypass times (*p* = 0.0615) in patients with higher BMI. BMI ≥25 was associated with an increased incidence of hospital-acquired pneumonia (*p* = 0.020) and new-onset atrial fibrillation (*p* = 0.036) compared to BMI <25. However, raised BMI did not extend ICU (*p* = 0.3310) or overall hospital stay (*p* = 0.2614). Similar rates of sternal wound complications, inotrope requirements and renal dysfunction were observed in both normal- and overweight/obese-BMI groups. Furthermore, increasing BMI correlated with reduced mechanical ventilation time (*p* = 0.039) and early post-operative blood loss (*p* = 0.004).

**Conclusions:**

Our results demonstrate that within the range of this study, MAAVR is a safe, reproducible and effective procedure, affording equivalent clinical outcomes in both overweight/obese and normal-weight patients considered for an isolated first-time AVR, with low post-operative morbidity and mortality. MAAVR should therefore be considered as an alternative surgical strategy to reduce obesity-related complications in patients requiring aortic valve replacement.

## Background

Surgical aortic valve replacement (AVR) via conventional median sternotomy has been established worldwide as a safe and feasible intervention for aortic valvular pathology [1]. Several minimal access strategies to access the aortic valve, including right para-sternal thoracotomy, partial upper hemi-sternotomy, transverse sternotomy, J-shaped partial upper sternotomy and complete median sternotomy via a limited skin incision [2–4] have been employed over the years with the aim of reducing surgical trauma. The proposed advantages of minimal-access aortic valve replacement (MAAVR) are reduced post-operative blood loss and transfusion requirements, reduced post-operative pain, shorter ventilation times, shorter length of intensive care unit and hospital stay, and enhanced cosmesis [2–6]. These benefits have been additionally validated in higher-risk elderly populations with multiple medical co-morbidities [7] who may tolerate prolonged operative and cardiopulmonary bypass times less well. However, obese patients are often denied MAAVR due to the perceived increase in technical difficulty associated with the procedure in such populations.

Over the past decade we have utilised a J-shaped partial upper sternotomy in ‘all comers’ as a first-line approach for MAAVR. In this retrospective analysis, we assess the effect of body mass index (BMI) on post-operative outcomes in unselected consecutive patients referred to a single surgeon for surgical AVR over a 7-year period.

## Methods

We performed a retrospective analysis of 90 patients who underwent elective isolated MAAVR through a J-shaped partial upper sternotomy between May 2006 and October 2013 under a single surgeon across three hospital sites (St Mary’s Hospital, Hammersmith Hospital, and The Wellington Hospital, London, UK). Patients with a severely-depressed left ventricular ejection fraction (<25 %), sternal deformity, heavy calcification of the ascending aorta, and concomitant coronary, mitral valve or tricuspid valve disease were excluded. One case was a re-operation. Data on pre-, intra-, and post-operative variables were collected retrospectively from the hospital cardiac surgical database. Pre-operative patient characteristics are shown in Table 1.

**Table 1:** Pre-operative patient characteristics

Variable	MAAVR patients (*n* = 90)
Age (years)	67.37 ± 15.46
Gender (male/female)	53/37
Height (cm)	167.2 ± 11.72
Weight (kg)	74.85 ± 14.38
Body mass index	26.63 ± 4.07
Hypertension (n)	42 (46.7 %)
Hypercholesterolaemia (n)	30 (33.3 %)
Diabetes (n)	9 (10 %)
Renal insufficiency (n)	1 (1.1 %)
COPD (n)	2 (2.2 %)
Cerebrovascular disease (n)	0 (0 %)
Previous PCI (n)	2 (2.2 %)
Ejection fraction (%)	57.31 ± 14.41
Aortic stenosis (n)	72 (80 %)
Aortic insufficiency (n)	13 (14.4 %)
Mixed stenosis/insufficiency (n)	5 (5.6 %)
Active endocarditis (n)	2 (2.2 %)
Logistic EuroSCORE	6.36 ± 5.5

Continuous variables expressed as mean ± SD

*COPD* chronic obstructive pulmonary disease, *EuroSCORE* European Score for Cardiac Operative Risk Evaluation, *MAAVR* minimal-access aortic valve replacement, *PCI* percutaneous coronary intervention

The surgical technique for aortic valve replacement via J-shaped partial upper sternotomy has been previously described [4]. A 6-7 cm midline skin incision is made over the upper sternum, followed by a limited upper sternotomy which extends in a “J” shape from the sternal notch to the right sternal edge at the third intercostal space. The sternum is spread to a maximum of 6 cm with a small sternal retractor to minimise stretching and avoid sternal fracture on the opposite side of the J incision. After pericardotomy, the aorta and right atrium are centrally cannulated in a routine manner via an EOPA® (Medtronic, Inc., USA.) aortic cannula and an Oval MC2™ (Medtronic, Inc., USA) double-stage venous cannula, respectively. Over the last 3 years in an increasing number of patients, a FlexFlow™ percutaneous double-stage venous cannula (Sorin Biomedica, Italy) has been positioned with its tip entering the superior vena cava under trans-oesophageal echocardiography guidance. An aortic root cannula is secured to the ascending aorta for antegrade cardioplegia delivery and venting/de-airing following aortic cross clamp removal. Cardiopulmonary bypass and cardioplegic arrest are instituted and maintained at moderate hypothermia (32 °C). Continuous carbon dioxide field-flooding is utilised to reduce the risk of gas embolisation. After application of an aortic cross-clamp via the sternal incision, a transverse aortotomy is made and aortic valve replacement performed in the standard fashion. The aortotomy is then repaired with a running suture and adequacy of de-airing with aortic root venting and/or needle aspiration is assessed on trans-oesophageal echocardiography. Temporary pacing wires are sited before removal of the aortic cross clamp and two 24Fr Blake® (Ethicon, USA) chest drains are placed via the right fifth intercostal space. The sternum is closed with steel wires. Post-operatively, all patients were transferred to the cardiothoracic intensive care unit, sedated and mechanically ventilated in accordance with local protocol. Follow-up with echocardiographic studies and clinical evaluation was performed at a mean of 2.5 years post-operatively (range 0.021–7.83 years).

Ordinary least squares univariate linear regression analysis was performed to determine the effect of BMI as a continuous variable on post-operative outcomes. One-way analysis of variance (ANOVA) was used to determine the effect of BMI as a categorical variable (factor 1: normal weight, BMI <25 vs. factor 2, overweight and obese, BMI ≥25) on continuous outcome variables. The Chi-squared statistic was used to determine the effect of the same BMI categories on binary outcome variables. A *p* value less than 0.05 was considered statistically significant. Data analysis was performed using StataMP version 12.1 (StataCorp, Texas).

## Results

Peri-operative outcomes and the correlation of these outcomes with BMI as both a continuous and categorical variable are shown in Tables 2 and 3 respectively. 28 % of patients undergoing MAAVR in our study were octogenarians, 51 % were aged over 70 years and 10 % were younger than 50 years. Mean BMI was 26.63 ± 4.07 (range 18.7–37.2 kg/m^2^), with 17 patients (19 %) having a BMI exceeding 30. Aortic valvular pathologies comprised stenosis in 72 patients, insufficiency in 13 patients and mixed disease in five patients. In 68 patients (76 %) a bioprosthesis was implanted, whilst 22 patients (24 %) received a mechanical prosthesis. The median size of implanted valves was 23 mm (range 19–25 mm).

**Table 2:** Peri-operative outcomes

Variable	MAAVR patients (*n* = 90)
Intra-operative conversion to full sternotomy (n)	0
Aortic cross-clamp time (min)	72.7 ± 14.9
CPB time (min)	88.1 ± 17.9
Biological prosthesis (n)	68
Mechanical prosthesis (n)	22
Median valve size (mm)	23 (19–25)
In-hospital mortality (n)	0
Mechanical ventilation time (hours)	6.51 ± 4.14
ICU stay (hours)	48.9 ± 28.9
Overall hospital stay (days)	8.68 ± 6.38
Bleeding at 12 h (ml)	469 ± 391
Re-exploration for bleeding (n)	2
RBC transfusion (n)	22
New-onset atrial fibrillation (n)	27
Respiratory tract infection (n)	4
Renal dysfunction (n)	4
Permanent pacemaker requirement (n)	2
Mechanical circulatory support (n)	1
Sternal instability (n)	0
Sternal wound infection (n)	0
Prosthetic valve endocarditis (n)	0
Myocardial infarction (n)	0
Cerebrovascular accident (n)	0

Continuous variables expressed as mean ± SD

*CPB* cardiopulmonary bypass, *ICU* intensive care unit, *MAAVR* minimal-access aortic valve replacement, *RBC* red blood cell

**Table 3:** Correlation between BMI and peri-operative outcomes. (a) Ordinary least squares univariate linear regression of BMI as a continuous variable; (b) Analysis of BMI as a categorical variable (factor 1 BMI <25 (*n* = 36) vs. factor 2 BMI ≥25 (*n* = 54)) using ANOVA (continuous dependent variables) and chi-squared (binary dependent variables) statistics

BMI as a continuous variable			
Variable	*r* ^*2*^	Coefficient	*p*
Aortic cross-clamp time	0.072	0.070	0.022
CPB time	0.043	0.045	0.062
Mechanical ventilation time	0.084	−0.295	0.039
ICU stay	0.019	0.941	0.331
Overall hospital stay	0.017	0.080	0.261
Bleeding at 12 h	0.067	−0.003	0.019
Bleeding prior to arrival on ICU	0.091	−0.010	0.031
RBC transfusion	0.034	−1.790	0.190
New-onset atrial fibrillation	0.067	−2.130	0.024
Hospital-acquired pneumonia	0.048	2.870	0.113
BMI as a categorical variable: normal (<25) vs. overweight-obese (≥25) patients			
Variable	f	Chi^2^	*p*
Aortic cross-clamp time	3.43	-	0.068
CPB time	4.93	-	0.029
Mechanical ventilation time	3.60	-	0.064
ICU stay	0.11	-	0.742
Overall hospital stay	0.17	-	0.678
Bleeding at 12 h	6.58	-	0.013
Bleeding prior to arrival on ICU	4.51	-	0.039
RBC transfusion	-	0.815	0.367
New-onset atrial fibrillation	-	4.410	0.036
Hospital-acquired pneumonia	-	5.400	0.020
Renal dysfunction	-	0.159	0.690

Continuous variables expressed as mean ± SD

*CPB* cardiopulmonary bypass, *ICU* intensive care unit, *MAAVR* minimal-access aortic valve replacement, *RBC* red blood cell; f- f statistic of ANOVA (ratio of the Mean Square Between (MSB) estimates to Mean Square Error (MSE) estimates)

### Intra-operative outcomes and length of stay

**Fig. 1:**
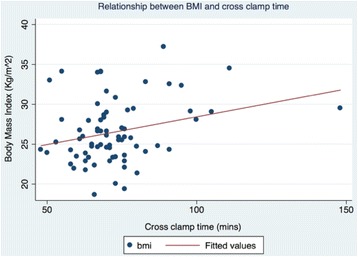
Univariate linear regression analysis of BMI against cross clamp time for MAAVR

There were no conversions to full sternotomy. Univariate regression demonstrated a significant positive correlation between BMI and cross-clamp time (*r*^*2*^ 0.072; coef. 0.070; *p* = 0.0218) (Fig. 1). However, BMI was not found to correlate significantly with overall cardiopulmonary bypass time (*r*^*2*^ 0.043; coef. 0.045, *p* = 0.0615).

A significant negative correlation was also observed between BMI and ventilation times (*r*^*2*^ 0.084; coef. -0.295; *p* = 0.039) however BMI did not correlate with post-operative inotrope usage (*r*^*2*^ 0.011; coef. 0.364; *p* = 0.359), intensive care unit (*r*^*2*^ 0.019; coef. 0.941; *p* = 0.3310) or overall hospital stay (*r*^*2*^ 0.017; coef. 0.080; *p* = 0.2614).

Analysis of BMI as a categorical variable (normal (BMI < 25) vs. overweight-obese (BMI ≥ 25) demonstrated a significantly longer overall cardiopulmonary bypass time (*p* = 0.029) in overweight-obese patients. No differences were observed in cross-clamp time (*p* = 0.068), ventilation time (*p* = 0.064), intensive care unit (*p* = 0.74) or overall hospital stay (*p* = 0.68) between normal and overweight-obese groups.

### Post-operative morbidity outcomes

There was no in-hospital myocardial infarction, stroke or mortality. One patient developed malignant arrhythmia immediately after coming off cardiopulmonary bypass, necessitating intra-aortic balloon pump insertion. The remainder of patients were successfully weaned off cardiopulmonary bypass without mechanical circulatory support.

One patient required full sternotomy for cardiac tamponade, and another was re-explored for bleeding but did not require extension of the original limited sternotomy. Two patients underwent permanent pacemaker implantation post-operatively, one for prolonged pacing dependence, and another for complete heart block.

A significant positive correlation was observed between BMI as a continuous variable and the incidence of post-operative atrial fibrillation (*r*^*2*^ 0.067; coef. -2.13; *p* = 0.0243). However, BMI was not significantly correlated with the incidence of renal dysfunction (*r*^*2*^ 0.004; coef. 1.12; *p* = 0.6551), or hospital-acquired pneumonia (*r*^*2*^ 0.048; coef. 2.87; *p* = 0.113). There were no cases of sternal dehiscence or infection.

Categorical analysis of normal vs. overweight-obese patients revealed a significantly higher incidence of AF (*p* = 0.036) and post-operative hospital acquired pneumonia (*p* = 0.020) in the overweight-obese group. However, no differences were observed in renal dysfunction (*p* = 0.69) between BMI categories.

**Fig. 2:**
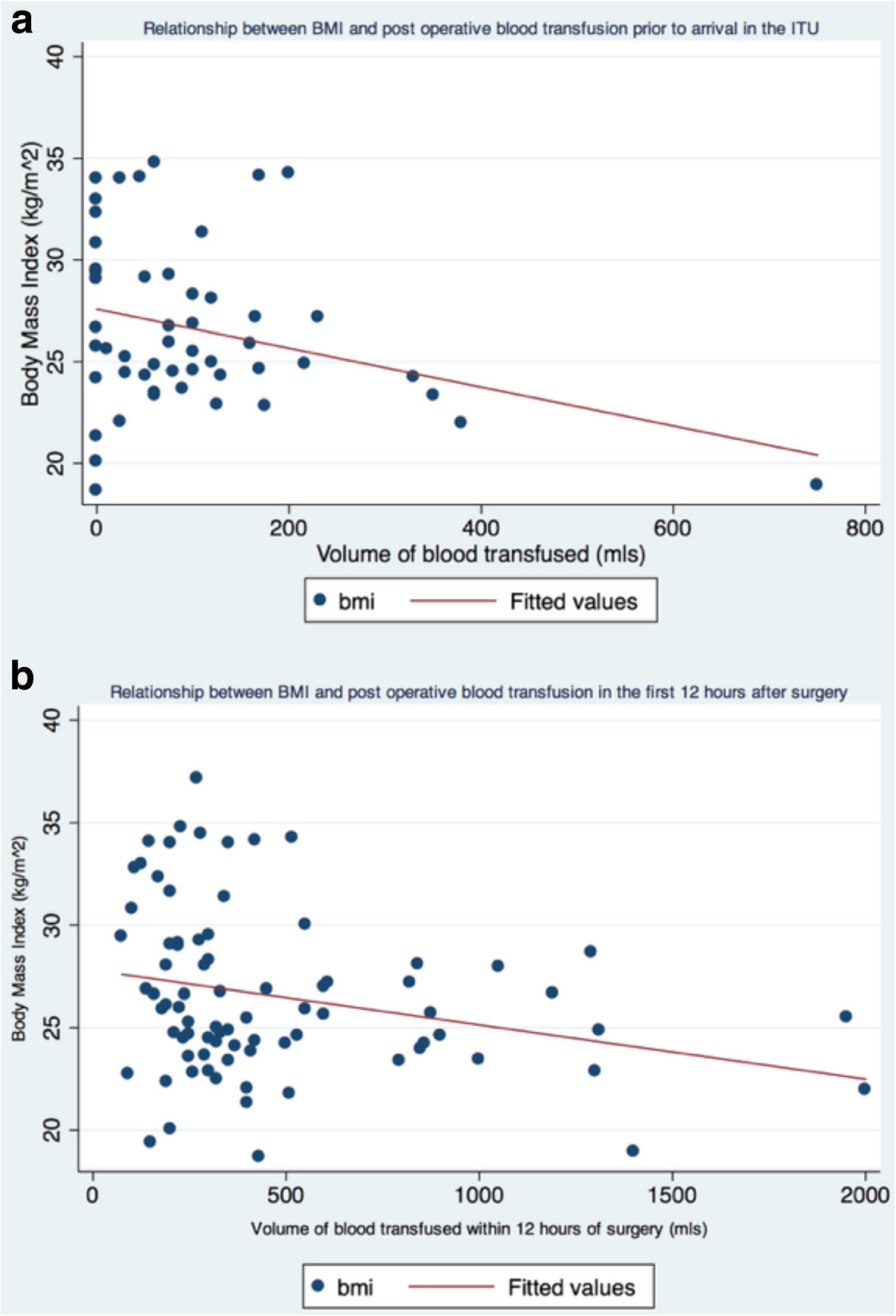
Univariate linear regression analysis of BMI against **a** peri-operative bleeding prior to arrival in ICU; **b** bleeding in the first 12 h post-operatively

Red blood cell transfusion was required in 21 patients (23 %). BMI demonstrated a significant negative correlation with bleeding in the immediate post-operative period prior to arrival on ITU (*r*^*2*^ 0.091; coef. -0.0096; *p* = 0.031) and within the first 12 h post-operatively (*r*^*2*^ 0.067; coef. -0.0027; *p* = 0.019) (Fig. 2a, b). However, this did not lead to any significant correlation between BMI and peri-operative transfusion rates (*r*^*2*^ 0.034; coef. -1.70; *p* = 0.1899). Similarly, categorical analysis of BMI revealed significantly less post-operative bleeding (prior to arrival in ITU) and 12-h blood loss (*p* = 0.039 and 0.013 respectively) in overweight-obese when compared to normal weight patients.

### Echocardiographic follow-up

Follow-up echocardiography performed in 35 patients at a mean 2.5 years (range 0.021–7.83 years) post-operatively demonstrated trivial para-valvular leak in one patient, mild para-valvular leak in four patients, and mild-moderate para-valvular leak in one patient. One patient with moderate para-valvular leak was re-operated via the same surgical approach 47 months after the first MAAVR. No patients required re-operation for prosthetic valve endocarditis.

## Discussion

Surgical AVR has traditionally been accomplished with excellent outcomes through a median sternotomy, permitting excellent exposure of the heart and great vessels [1]. This relatively invasive incision may however produce significant post-operative pain, impairment of respiratory function [8, 9], potential for chest wall instability, wound infection and a visually unattractive scar.

**Fig. 3:**
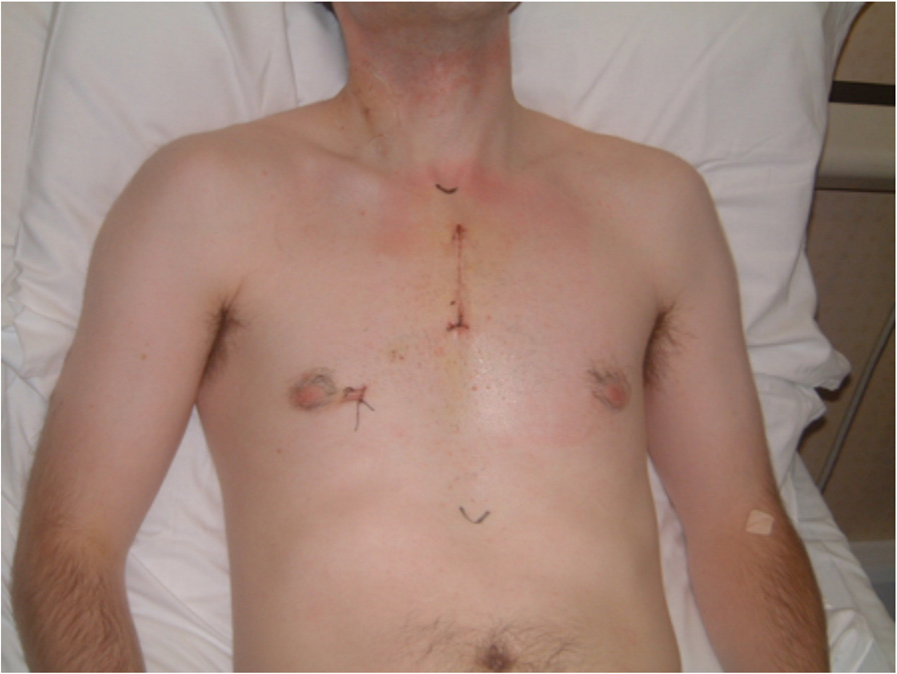
Post-operative scar following J-shaped partial upper sternotomy for MAAVR

Thus, over the past two decades, several minimal access approaches for AVR have been introduced with variable uptake [2–4]. These have the potential advantages of less post-operative pain, decreased blood loss, shorter ventilation times, shorter durations of intensive care unit and hospital stay, faster functional recovery and favourable cosmesis [2–6] (Fig. 3). Despite these benefits, several meta-analyses have indicated no significant morbidity or mortality difference for patients undergoing either conventional or minimally-invasive AVR [10], and there is little evidence supporting MAAVR in obese patients.

We sought to determine the effect of BMI on outcomes in MAAVR performed via J-shaped partial upper sternotomy. Our results demonstrate that within the range of this study MAAVR can be safely performed in patients of higher BMI considered for an isolated first-time AVR with low post-operative morbidity and mortality.

Our findings demonstrate that MAAVR in patients with a higher BMI does not lead to prolonged length of intensive care or overall hospital stay. Furthermore, although we observed a significant positive correlation between BMI and cross-clamp time this was not reflected in total cardiopulmonary bypass times. Indeed, cross-clamp times were generally similar to those reported by other groups, who have not demonstrated these to be significantly longer with minimally invasive incisions [6, 11, 12]. It can also be expected that once the initial aspect of the learning curve has been negotiated, operative times in MAAVR may further trend downwards with accruing surgical experience. In addition, with the more recent adoption of rapid-deployment ‘sutureless’ aortic prostheses is likely we will see a further decrease aortic cross-clamp and cardiopulmonary bypass times [13].

New-onset atrial fibrillation remains a pertinent issue after MAAVR, reported in 22–34 % of minimally invasive cohorts analysed by other groups [2, 11, 14, 15]. In keeping with the findings of previous studies [16, 17], our results demonstrated a significant positive correlation between new onset post-operative atrial fibrillation and BMI. Although the exact mechanism for this is likely to be multifactorial, it has been postulated that left atrial enlargement as a result of increasing BMI plays a key role in the setting up and maintenance of atrial fibrillation re-entrant circuits [16]. Indeed, our results demonstrate a highly-significant positive correlation between pre-operative left atrial size and increasing BMI (*r*^*2*^ 0.2353; coef. 2.92; *p* = 0.001), suggesting that obesity-associated left atrial enlargement may present a quantifiable risk factor for the development of new-onset atrial fibrillation after MAAVR.

No significant correlation was found between BMI and other post-operative morbidities suggesting that within the range studied here, increasing BMI should not contraindicate a minimally invasive approach. Furthermore, there were no deaths in our series over the follow-up period, supporting evidence from other studies that MAAVR is associated with similar mortality rates to standard AVR [1, 3, 8, 9, 11, 15, 18–25].

Using this less-invasive approach comprising only partial sternotomy, a smaller area of exposed sternal bone marrow and minimised mediastinal dissection should theoretically produce less bleeding and ultimately improved sternal healing. Indeed, significantly reduced haemorrhage and blood product utilisation have been reported with MAAVR techniques [6, 15], with acceptable rates of re-operation for bleeding compared to conventional AVR [14]. In our experience no patients required intra-operative conversion to median sternotomy. Red blood cell transfusion was indicated in 26 % of patients in the present series with only two patients (2.2 %) requiring surgical re-exploration. We did not observe any increase in post-operative blood loss or transfusion requirement with increasing BMI, and, in fact, a significant negative correlation was seen between BMI and bleeding in the first 12 h following surgery. Furthermore, we observed no cases of sternal dehiscence. This highlights that with meticulous haemostasis, low rates of intra-operative blood loss may be achieved with MAAVR, independent of BMI. It is also notable that in the event of haemorrhage or technical difficulties conversion to full median sternotomy can still be carried out expediently.

### Limitations

Our study is not without several important limitations. Firstly, it was a retrospective, non-comparative observational study comprising a relatively small group of consecutive patients referred for surgical AVR. As such, no direct outcomes comparison of MAAVR to conventional AVR was possible. Secondly, although all operations were conducted by a single surgeon, this was across three different institutions, where slight variations in peri-operative hospital protocols may exist. Thirdly, only 19 % of our patients were obese (BMI > 30) according to WHO criteria, and therefore overweight and obese patients were pooled into a single group for statistical comparison. Further work would require a larger sample population of patients with BMI > 30 to allow this group to be considered separately. Finally, our study lacks evaluation of patient satisfaction outcome measures, such as post-operation pain and quality of life, which are pertinent aspects of a minimal access procedure. We also did not perform any economic analysis to assess the financial implications of MAAVR in relation to standard AVR, since this was beyond the scope of our study.

In addition, there are a number of technical considerations that must be taken into account when adopting this minimal access technique. Although the use of a J-shaped partial sternotomy removes the requirement for pre-operative CT scanning to establish the aortic annular position, as required for approach through a second intercostal right mini-thoracotomy, it confers some technical difficulties. Firstly, placement of epicardial pacing wires is more demanding, and has to be performed before removal of the aortic cross clamp in the arrested heart. Second, internal defibrillation is not possible and external defibrillator pads must be applied prior to operation in all patients. Furthermore, removal of air from the heart after completion of the surgical procedure has been cited as a particular concern [12, 18], and, as such, to achieve satisfactory de-airing we employ continuous carbon dioxide field insufflation and aortic root venting or needle aspiration under direct trans-oesophageal echocardiographic monitoring.

## Conclusion

Our 7-year data presented here support J-shaped partial upper sternotomy as a safe, reproducible and effective approach for MAAVR, offering good short- to mid-term outcomes in normal, overweight and obese patients. Further randomised study of obese patients, specifically with a BMI > 30 kg/m^2^ is now required to assess the role of MAAVR when compared to conventional sternotomy in this patient group who until now have been considered unsuitable for such a minimal-access approach.

## Ethical approval

Not required.

## Consent

Written informed consent was obtained from the patient for the publication of the image included in this manuscript.

## Abbreviations

AVR: aortic valve replacement
BMI: body mass index
COPD: chronic obstructive pulmonary disease
CPB: cardiopulmonary bypass
ICU: intensive care unit
MAAVR: minimal access aortic valve replacement
PCI: percutaneous coronary intervention
RBC: red blood cells

## References

[1] von Segesser LK, Westaby S, Pomar J, Loisance D, Groscurth P, Turina M (1999). Less invasive aortic valve surgery: rationale and technique. Eur J Cardiothorac Surg.

[2] Doll N, Borger MA, Hain J, Bucerius J, Walther T, Gummert JF, Mohr FW (2002). Minimal access aortic valve replacement: effects on morbidity and resource utilization. Ann Thorac Surg.

[3] Raja SG, Benedetto U (2013). Minimal access aortic valve replacement via limited skin incision and complete median sternotomy. J Thorac Dis.

[4] Raja SG, Benedetto U, Amrani M (2013). Aortic valve replacement through J-shaped partial upper sternotomy. J Thorac Dis.

[5] Tabata M, Umakanthan R, Cohn LH, Bolman RM, Shekar PS, Chen FY, Couper GS, Aranki SF (2008). Early and late outcomes of 1000 minimally invasive aortic valve operations. Eur J Cardiothorac Surg.

[6] Bonacchi M, Prifti E, Giunti G, Frati G, Sani G (2002). Does ministernotomy improve postoperative outcome in aortic valve operation? A prospective randomized study. Ann Thorac Surg.

[7] Schmitto JD, Mohr FW, Cohn LH (2011). Minimally invasive aortic valve replacement: how does this perform in high-risk patients?. Curr Opin Cardiol.

[8] Alassar Y, Yildirim Y, Pecha S, Detter C, Deuse T, Reichenspurner H (2013). Minimal access median sternotomy for aortic valve replacement in elderly patients. J Cardiothorac Surg.

[9] Chang YS, Lin PJ, Chang CH, Chu JJ, Tan PP (1999). “I” ministernotomy for aortic valve replacement. Ann Thorac Surg.

[10] Murtuza B, Pepper JR, Stanbridge RD, Jones C, Rao C, Darzi A, Athanasiou T (2008). Minimal access aortic valve replacement: is it worth it?. Ann Thorac Surg.

[11] Aris A, Camara ML, Montiel J, Delgado LJ, Galan J, Litvan H (1999). Ministernotomy versus median sternotomy for aortic valve replacement: a prospective, randomized study. Ann Thorac Surg.

[12] Cooley DA (1998). Minimally invasive valve surgery versus the conventional approach. Ann Thorac Surg.

[13] Rubino AS, Santarpino G, De Praetere H, Kasama K, Dalen M, Sartipy U, Lahtinen J, Heikkinen J, Deste W, Pollari F, Svenarud P, Meuris B, Fischlein T, Mignosa C, Biancari F (2014). Early and intermediate outcome after aortic valve replacement with a sutureless bioprosthesis: Results of a multicenter study. J Thorac Cardiovasc Surg.

[14] Cohn LH, Adams DH, Couper GS, Bichell DP, Rosborough DM, Sears SP, Aranki SF (1997). Minimally invasive cardiac valve surgery improves patient satisfaction while reducing costs of cardiac valve replacement and repair. Ann Surg.

[15] Bakir I, Casselman FP, Wellens F, Jeanmart H, De Geest R, Degrieck I, Van Praet F, Vermeulen Y, Vanermen H (2006). Minimally invasive versus standard approach aortic valve replacement: a study in 506 patients. Ann Thorac Surg.

[16] Zacharias A, Schwann TA, Riordan CJ, Durham SJ, Shah AS, Habib RH (2005). Obesity and risk of new-onset atrial fibrillation after cardiac surgery. Circulation.

[17] Wang TJ, Parise H, Levy D, D’Agostino RB, Wolf PA, Vasan RS, Benjamin EJ (2004). Obesity and the risk of new-onset atrial fibrillation. JAMA.

[18] Machler HE, Bergmann P, Anelli-Monti M, Dacar D, Rehak P, Knez I, Salaymeh L, Mahla E, Rigler B (1999). Minimally invasive versus conventional aortic valve operations: a prospective study in 120 patients. Ann Thorac Surg.

[19] Szwerc MF, Benckart DH, Wiechmann RJ, Savage EB, Szydlowski GW, Magovern GJ, Magovern JA (1999). Partial versus full sternotomy for aortic valve replacement. Ann Thorac Surg.

[20] Liu J, Sidiropoulos A, Konertz W (1999). Minimally invasive aortic valve replacement (AVR) compared to standard AVR. Eur J Cardiothorac Surg.

[21] Paredes FA, Canovas SJ, Gil O, Garcia-Fuster R, Hornero F, Vazquez A, Martin E, Mena A, Martinez-Leon J (2013). Minimally invasive aortic valve surgery. A safe and useful technique beyond the cosmetic benefits. Rev Esp Cardiol (Engl Ed).

[22] Christiansen S, Stypmann J, Tjan TD, Wichter T, Van Aken H, Scheld HH, Hammel D (1999). Minimally-invasive versus conventional aortic valve replacement--perioperative course and mid-term results. Eur J Cardiothorac Surg.

[23] Gilmanov D, Bevilacqua S, Murzi M, Cerillo AG, Gasbarri T, Kallushi E, Miceli A, Glauber M (2013). Minimally invasive and conventional aortic valve replacement: a propensity score analysis. Ann Thorac Surg.

[24] Johnston DR, Atik FA, Rajeswaran J, Blackstone EH, Nowicki ER, Sabik JF, Mihaljevic T, Gillinov AM, Lytle BW, Svensson LG (2012). Outcomes of less invasive J-incision approach to aortic valve surgery. J Thorac Cardiovasc Surg.

[25] Masiello P, Coscioni E, Panza A, Triumbari F, Preziosi G, Di Benedetto G (2002). Surgical results of aortic valve replacement via partial upper sternotomy: comparison with median sternotomy. Cardiovasc Surg.

